# The ISAC Paradigm to Tame Oral Cancer in Saudi Arabia: A Quasi-experimental Study

**DOI:** 10.1007/s13187-023-02356-y

**Published:** 2023-08-18

**Authors:** Mohammed Jafer, Ibtisam Moafa, Ciska Hoving, Math Candel, Abdulrahman A. Kaabi, Bart Van Den Borne

**Affiliations:** 1 Dental Public Health Division, Department of Preventive Dental Sciences, Jazan University, Jazan, Saudi Arabia; 2 Department of Health Promotion, Care and Public Health Research Institute (CAPHRI), Maastricht University, Maastricht, The Netherlands; 3 Department of Research and Methodology, Care and Public Health Research Institute (CAPHRI), Maastricht University, Maastricht, The Netherlands; 4 Community Service Unit, College of Dentistry, Jazan University, Jazan, Saudi Arabia

**Keywords:** Early detection, Screening, Mouth neoplasm, Public health, Behavior change, Health education, Cancer prevention, Psychological health

## Abstract

Late detection of oral cancer (OC) cases in Saudi Arabia is concerning. It reduces survival rate and complicates treatment. The ISAC intervention was developed to bridge the gaps observed in dentists’ practice of OC examination and patient education. The ISAC stands for I, informing patients of OC screenings; S, screening for OC; A, advising high-risk patients to quit risk factors; and C, connecting patients to advanced services. This study tested the potential effect of the ISAC in influencing dentists’ cognitive and behavioral skills, to enhance early detection and prevention of OC. A quasi-experimental study was conducted among dental interns (DIs) at dental setting to test the effect on comprehensive oral cancer examination score (COCE), awareness, self-efficacy, descriptive-norms, and self-reported behavior. Data were collected through triangulation of methods pre and post the intervention at two-months. Multiple linear mixed effects regression models were utilized for data analysis. Between October 2020 and April 2021, 47 DIs participated in the study. The final model showed the significant effects of time (ISAC) on COCE (95% CI = 25.12–29.42, *P* < .001). DIs had a significant improvement in awareness, self-efficacy, descriptive norms, and self-reported behavior. The findings showed promising effects of the intervention toward the early detection and prevention of OC. Dentists, dental organizations, and policymakers in areas with a high risk of OC could benefit from the current intervention which contributes to capacity building and improved community health. A pragmatic study with a robust design is needed to test the effectiveness of the intervention on a wider scale.

## Introduction

Oral cancer (OC) is a public health problem that puts a significant burden on developing countries [1–3]. Among the Arabian Gulf countries, Saudi Arabia has the highest age-standardized death rates associated with OC (ASMR = 1.74) compared to other types of cancer [4]. Frequent use of “Shammah” is commonly associated with OC cases in Saudi Arabia [4, 5]. Shammah is a local type of smokeless tobacco (ST) that is placed between the teeth and the inner surface of cheeks or below the tongue for a few minutes, and then it is spat out [5]. Shammah is made of ground tobacco leaves, ground glass, pepper, and coloring and flavoring agents [5]. Globally, the use of ST accounted for the death of around 315,000 people in 2016 [6, 7]. However, there is neither sufficient published information about the prevalence of Shammah use, nor about the death rate consequential to its use in Saudi Arabia.

The high numbers of OC cases detected at a late stage in the Jazan region of Saudi Arabia are concerning [5, 6]. OC late-stage is associated with a poor prognosis and complex treatments [5, 6]. OC late detection can be linked to patients’ factors, healthcare system’s factors, and dentists’ factors [8, 9]. From a patient’s perspective, a lack of awareness about OC and low access to healthcare services was commonly associated with OC late detection [10, 11]. From the perspective of the healthcare system, referral patterns and longer waiting times for appointments were found to adversely affect OC treatment options [12], whereas the third angle is related to dentists’ negative behavior and perceptions which further exacerbate OC late detection [13]. Previous evidence revealed a lack of dentists’ performance of OC examination and patient education practice [13]. Likewise, multiple studies in the Jazan region revealed a lack of OC examinations and patient education among dentists, dental interns, and dental students [9, 14, 15]. The findings of these studies were statistically and narratively consistent with the information collected from dental patients and tobacco users in the same region [13]. Knowledge, low-confidence, deficient screening skills, passive descriptive norms towards OC examination, inadequate clinical time for OC screening, and preference of stabilizing previously existing conditions, as well as OC screening costs, were considered the determinants that are high likely to be associated with the observed lack of OC examination and patient education [16]. In addition, these studies revealed that dentist’s gender perceived to be a barrier against performing comprehensive OC examinations and educating patients [9, 16]. Previous evidence has revealed the beneficial effect of dentists practicing OC examinations and patient education on lessening the mortality rate in high-risk people [3, 17]. However, none of the previous interventions have been developed based on the country’s specific needs or guided by an evidence-based approach to curbing OC in Saudi Arabia. Most of the existing community outreach programs have been organized by volunteering dental students and did not rely on a sound evidence-based approach [18]. Moreover, these activities were limited and focused only on raising public knowledge and awareness of OC in the region’s population. A previous intervention was conducted in 2014 to improve young people’s knowledge of OC in the Jazan region [18]. The intervention components included a lecture, an educational brochure, and a question-answer session delivered only once and at a single point in time. The intervention reported a significant improvement in the participants’ knowledge and awareness of OC and its harmful effects [18]. However, it should also be noted that although knowledge might be a necessary first step, it is insufficient to lead to behavior change [19, 20]. Moreover, the intervention did not include any measurements to assess participants’ self-efficacy and behavior. The fact that this intervention did not include methods to increase motivation or skills training could also have elicited adverse effects in the young people’s response to the provided health educational messages. According to the fear appeal theory, this adverse effect is a defensive avoidance response [21]. Therefore, the ISAC protocol was developed using the intervention mapping approach in an iterative process by a multidisciplinary team of researchers, stakeholders and representatives to improve four main behavioral goals: I = informing dental patients about performing routine OC screenings; S = screening for OC according to the clinical guidelines; A = advising patients who are at high risk to quit risk factors and perform OC self-examinations, and C = connecting patients to advanced and specialized services if necessary [22]. These four behavioral goals cover major issues in dentists’ behavior found from the needs assessment, as it was shown that dentists were not informing patients about OC examination, not performing complete OC examinations, not educating patients on OC risk factors, and demonstrating deficient communication skills with patient, and they did not connect patients to specialized services when they required advanced help. The intervention protocol and the usability pretesting were recently published and can be accessed in < ISACprotocol > and < PretestingISAC > .

Tailoring health interventions to the particular context and the target group is highly recommended by previous research to increase the acceptability and adoption of health interventions [23]. Following the intervention pretesting and after assessing its acceptability, compatibility with existing norms/values, feasibility, and having performed the necessary refinements [24], this study is aimed at implementing and evaluating the effect of the intervention. The evaluation helps to assess whether the intervention had influenced the determinants and produced the expected outcomes [22, 25]. An effect evaluation of the ISAC intervention is integral to understanding the changes resulting from the intervention in a particular population and certain circumstances. The findings from this study will assist in informing future decision-making on whether to improve, reorient, discontinue, or scale up the intervention.

In the current stage, the study targeted Saudi dental interns (DIs) from the same academic year as they are the first to examine the patients in Jazan Dental School (JDS)-clinics and they scored the lowest in performing OC examination and patient education. The JDS-clinics is the largest dental institution in the Jazan region with 80 annual graduates serving thousands of patients. Therefore, the aim of the present study was to assess the potential effect of the ISAC intervention on the DIs perceptions and behavioral skills to prevent and detect OC at its early stage. We also examined as to whether the intervention effect differed for DIs based on gender (male or female). The alternative hypothesis was as follows: The ISAC intervention has an effect on dental interns’ perceptions and skills regarding early detection and prevention of oral cancer, while the null hypothesis was as follows: The ISAC intervention has no effect on dental interns’ perceptions and skills regarding early detection and prevention of oral cancer.

## Methods

### Study Design and Setting

We performed a six-month quasi-experimental study (an interrupted time-series design) to assess the ISAC intervention’s effect on dental interns’ (DIs) cognitive and behavioral skills. The research ethics committee at Jazan University (Registry No. [CDREC-06], dated 21 December 2016) reviewed and accepted the study protocol as part of an ongoing OC project. The present study was conducted in line with the Declaration of Helsinki. The intervention started in October 2020 and finished at the end of March 2021 at the setting of JDS-clinics. We utilized a convenience sample—the existing dental interns at JDS (*n* = 50)—because it was unfeasible to draw a random sample. Assigning DIs into two groups would negatively affect the study power and would lead to a high contamination between the study groups. DIs were invited to the study through university e-mail, and all of them have provided a written consent.

### Intervention

**Fig. 1:**
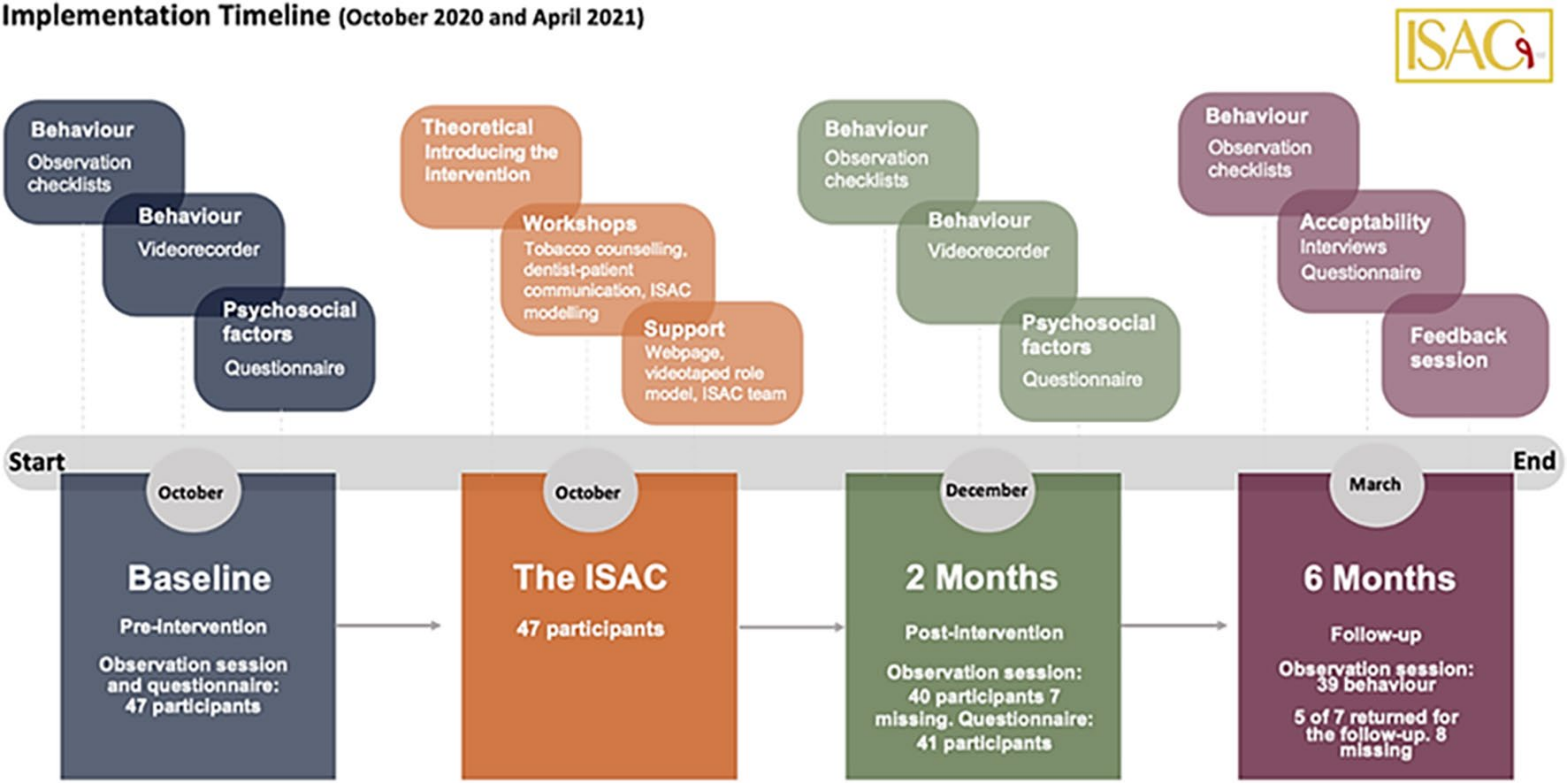
The timeline sequence of the intervention and the activities per measurement

The delivery of the ISAC intervention started with a PowerPoint session included an introduction to OC screening, its relevance and importance, introducing the ISAC concept, OC epidemiology, tobacco counseling, patient education, and connecting patients to specialized centers (Fig. 1).

Two face-to-face workshops of multiple sessions were conducted following the ppt by experts in tobacco counseling and patient communication skills. After these workshops, a final workshop was delivered on practicing the ISAC (covering all previous elements) with the use of role modeling and feedback on the DIs’ performance.

To reinforce the DIs’ practice of the ISAC, the practice guideline and its modeling video were uploaded on a specially developed webpage for the ISAC intervention. The ISAC practice was modeled by a highly skillful DI, who was identified as a role model among the other DIs, to ease the perceived relevance and identification.

### Procedures and Measurements

Primary outcome included comprehensive oral cancer examination (COCE) score and was assessed prior to the intervention, two months after the intervention, and at six months using a 29-item checklist. The checklist was a partially modified version of a published observation checklist developed and internally validated by Jafer et al., to assess dentists’ behavior toward COCE [9]. Further, it was weighted according to the items’ relevance and importance, as reported in the current literature. The higher the score, the better the performance (minimum score = 3, maximum score = 55). The observation checklist and the justification of the selected items are available at the following link: < ObservationChecklistsISAC > . After each measurement, a feedback discussion was conducted to reflect on the DIs’ performance and investigate any possible challenges or barriers during their practice of the ISAC (Fig. 1). The DIs’ performance of COCE was videotaped (during the performance) for 15 DIs (who had agreed to be recorded) as an indirect measurement to validate the findings that were obtained by the observer. After each data collection phase, one author from the intervention developers watched the video records and filled in the observation checklist.

Secondary outcomes included DIs’ awareness, self-efficacy, descriptive norms, and their self-reported behavioral skills, and were assessed using a self-reporting questionnaire (SR-Questionnaire). The SR-Questionnaire assessed the constructs through five-point Likert answer’s scales and ranged from 1 (not at all) to 5 (highly) for self-efficacy; 1 (extremely unlikely) to 5 (extremely likely) for descriptive norms; and 1 (never) to 5 (always) for self-reported behavioral skills. Awareness was assessed with 10 true-or-false questions, using the total score of correctly answered questions, with ten representing the highest awareness level. We added the Social Desirability Scale (SDS) to the SR-Questionnaire to adjust for the potential influence of social desirability on the DIs’ responses to the questionnaire [26, 27]. SDS coded 0 (not a sociably-desirable answer) and 1 (socially-desirable answer), and then the total score of the given social desirability answers was recorded for each participant, with ten representing the highest social desirability tendency. We utilized a 10-item (X1/M-C1) version of the Marlowe-Crowne SDS, which was developed by Strahan and Gerbasi and has a high level of internal consistency [26]. The SR-Questionnaire is available at < OCpracticeQues/ISAC > . The SR-Questionnaire and the 29-items checklist were evaluated for readability, comprehension, appropriateness, and suitability, using think-aloud among 15 participants (three dentists from the Ministry of Health-clinics, two dentists from private clinics, four dentists from JDS, and six dental interns).

### Statistical Analysis

All statistical analyses performed using IBM SPSS Statistics for Mac, version 26. The intraclass correlation coefficient (ICC) for the agreement between the observer and patient’s checklists was 0.998, with 95% CI ranging from 0.997 to 0.999. The ICC for the agreement between the observer, patient, and scores obtained from 15 video-recorded participants was 0.979, with 95% CI ranging from 0.914 to 0.994. Internal consistency Cronbach’s *α* were *α* = 0.862, 95% CI = 0.79 to 0.91, *α* = 0.875, 95% CI = 0.81 to 0.92, and *α* = 0.802, 95% CI = 0.70 to 0.87 for self-efficacy, descriptive norms and self-reported behavioral skills respectively. We checked the normality of outcome variables using the Shapiro-Wilk test and histograms at each time point and visually compared the trend in the average COCE score over the intervention period between male and female DIs. For the effect of the ISAC, we performed multiple regression analyses using two-level linear mixed effects model to account for missing data, dental interns’ heterogeneity, and repeated measurements within the same DI. The three measurement time points were coded through two dummy variables, with the baseline measurement as the reference category. We firstly investigated whether the variance-covariance structure of the random effects could be simplified. Secondly, we chose the appropriate structure with the lowest value for Akaike Information Criterion and Bayesian Information Criterion. Thirdly, we examined the fixed part of the model through a top-down approach by removing the least significant and non-significant interactions step by step. The final model’s fixed part included time, SDS, gender of DIs, and outcome variables. We adhered to the STROBE guidelines in reporting the intervention findings [28].

## Results

There were 47 Saudi DIs in the same academic year, and the same age group (25–27 years) at Jazan University participated in the study. Seven DIs missed the post-intervention measurement at two months. Two of them returned for the third evaluation period at six months. The reason of their absences was that they were traveling (Table 1).

**Table 1:** The characteristics of participants throughout the intervention period of six months

Performance related to COCE*	Male	Female	Total
Baseline	22	25	47
2 months post-intervention	16	24	40
6 months follow-up	18	21	39
ISAC Questionnaire	Male	Female	
Baseline	22	25	47
2 months post-intervention	21	20	41

*COCE comprehensive oral cancer examination practice

**Fig. 2:**
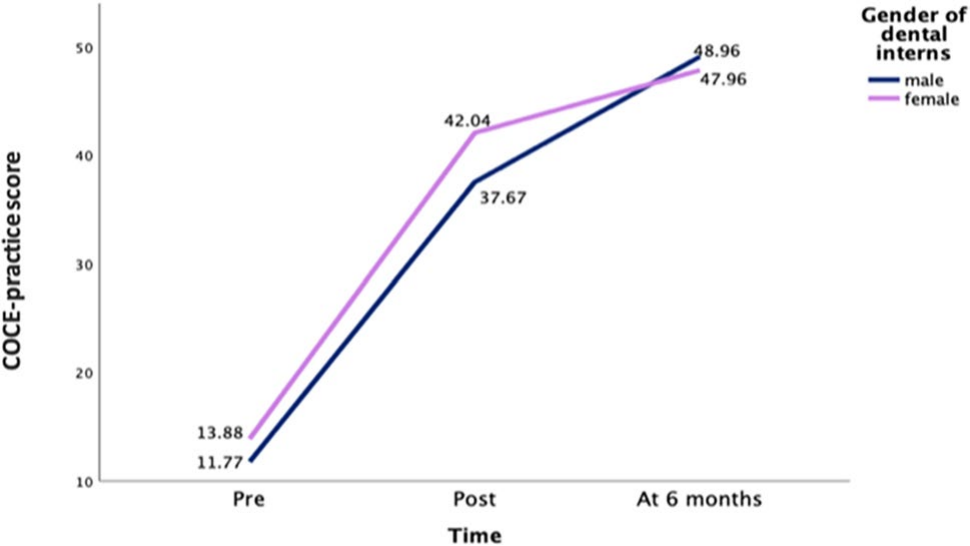
Mean profiles of comprehensive oral cancer examination score (COCE) of male and female dental interns over time

In the mean profile plot (Fig. 2), where we explored the COCE score per gender, female DIs tended to score relatively higher than males at both baseline and post intervention. In contrast, male DIs had slightly higher COCE scores than females at the six-month follow-up.

### The ISAC’s Effect on Primary Outcome (COCE)

The marginal model with heterogeneous compound symmetry variance-covariances was found to be superior (BIC = 773.504, AIC = 734.714) to other models with different structures of variance-covariances of the random effects. The final linear mixed effects model showed the significant effects of time at two months (*β* = 27.37, 95% CI = 25.12–29.42, *P* < 0.001), at six months (*β* = 35.22, 95% CI = 32.76–37.66, *P* < 0.001), and the gender of DIs on COCE (*β* = 2.25, 95% CI = 0.47–4.02, *P* = 0.014) (Table 2). On average, the COCE score improved two months after the intervention and at the six-month follow-up, as compared to the baseline rate. Aside from this, on average, females scored higher than males as shown in Fig. 2, but we found no significant interaction between the time dummies and gender based on the current data. Therefore, the effect of the intervention was the same for the male DIs as for female DIs (Table 2).

**Table 2:** Results of the linear mixed effects model analyses* examining the effect of the ISAC intervention on dental interns’ practices of the comprehensive oral cancer examination, awareness, self-efficacy, descriptive norms, and self-reported behavior

Primary outcome: comprehensive oral cancer practice score (*n* = 47)				
	*β*	Standard error	95% confidence interval	*P*-value
Intercept	10.54	1.76	7.02 to 14.05	** < 0.001**
Post-intervention**	27.26	1.07	25.12 to 29.42	** < 0.001**
Follow-up**	35.22	1.22	32.76 to 37.67	** < 0.001**
Gender of dental interns	2.25	0.89	0.47 to 4.03	**0.014**
Social desirability	0.27	0.23	− 0.18 to 0.73	0.229
Secondary outcome: awareness score (*n* = 41)				
	*β*	Standard error	95% confidence interval	*P*-value
Intercept	6.73	0.313	6.09 to 7.35	** < 0.001**
Post-intervention	3.07	0.24	2.59 to 3.55	** < 0.001**
Gender of dental interns	0.08	0.18	− 0.28 to 0.45	0.64
Social desirability	− 0.12	0.04	− 0.20 to − 0.03	**0.006**
Secondary outcome: self-efficacy score (*n* = 41)				
	*β*	Standard error	95% confidence interval	*P*-value
Intercept	26.63	1.92	22.76 to 30.49	** < 0.001**
Post-intervention	3.79	1.24	1.28 to 6.29	**0.004**
Gender of dental interns	2.13	1.14	− 0.17 to 4.43	0.307
Social desirability	0.27	0.26	− 0.26 to 0.82	0.069
Secondary outcome: descriptive norms score (*n* = 41)				
	*β*	Standard error	95% confidence interval	*P*-value
Intercept	19.7	2.72	14.30 to 25.14	** < 0.001**
Post intervention	14.04	3.73	6.49 to 21.57	**0.001**
Gender of dental interns	1.44	1.27	− 1.14 to 4.02	0.266
Social desirability	− 1.01	0.42	− 1.85 to − 0.16	**0.019**
Post-intervention *social desirability	− 2.10	0.58	− 3.29 to − 0.92	**0.001**
Secondary outcome: descriptive norms (*n* = 41) at baseline				
Social desirability	1.09	0.41	0.27 to 1.92	**0.010**
Secondary outcome: descriptive norms (*n* = 41) at post-intervention				
Social desirability	− 1.01	0.42	− 1.86 to − 0.17	**0.019**
Secondary outcome: self-reported behavior score (*n* = 41)				
	*β*	Standard error	95% confidence interval	*P*-value
Intercept	26.72	1.87	22.96 to 30. 47	** < 0.001**
Post-intervention	2.90	1.28	0.31 to 5.48	**0.029**
Gender of dental interns	0.40	1.12	− 1.81 to 2.63	0.712
Social desirability	0.35	0.25	− 0.16 to 0.87	0.174

*The statistical analyses were conducted using the following regression model: a linear mixed effects model for multilevel analysis of longitudinal data

**Preintervention is the reference point for post-intervention and follow-up

### The ISAC’s Effect on Secondary Outcomes

The marginal models with a compound symmetry covariance structure were found to be superior to the other structures for each of the four secondary outcomes. The final four linear mixed effects models showed that, on average, DIs had a significant improvement in awareness (*β* = 3.07, 95% CI = 2.59 to 3.55, *P* < 0.001), self-efficacy (*β* = 3.79, 95% CI 1.28 to 6.29, *P* = 0.004), descriptive norms towards OC examinations and patient education (*β* = 14.04, 95% CI = 6.49 to 21.57, *P* < 0.001), and self-reported behavior scores (*β* = 2.90, 95% CI = 0.31 to 5.48, *P* = 0.029) at two months after the intervention, compared to the baseline (Table 2). A significant negative interaction was observed between SDS and the two-month dummy variable in the descriptive norms model. We performed further effects analysis for the SDS at baseline and post-measurement. At the baseline, the effect of SDS on descriptive norms was found to be significantly positive (*β* = 1.09, 95% CI = 0.27 to 1.92, *P* = 0.010), but it was significantly negative at the post-measurement stage (*β* = − 1.01, 95% CI = − 1.86 to − 0.17, *P* = 0.019) (Table 2).

## Discussion

This study was aimed at assessing the potential effect of practicing the ISAC intervention in influencing dental interns’ (DIs’) perceptions and behavioral skills as well as whether the intervention effect differed for DIs based on gender. The intervention showed significant improvements in DIs’ perceptions and skills toward OC examinations and patient education, which agrees with the findings from the ISAC usability pretesting study [24]. Further, there was a significant effect of DIs gender on COCE, female DIs tend to score relatively higher than males at both baseline and post intervention. Which is consistent with the findings from previous two qualitative studies in which participating dentists and DIs believed that dentist’s gender influences the COCE practice [16, 24]. Nevertheless, it opposes the findings from the clinical observational study that did not find significant gender differences on COCE [9].

DIs’ perceptions and skills toward OC examination and patient education are vital to increase the early detection and prevention of OC. The positive changes found in DIs’ awareness, descriptive norms, self-efficacy, and self-reported behavior after the ISAC intervention was associated with observed positive behavior change in DIs’ practice of OC examination and patient education, which is consistent with previous studies’ findings that linked the influence of the negative beliefs in self-efficacy, descriptive norms and lack of skills to the non-practice of OC examination and patient education in the Jazan region [9, 16]. In addition, it agrees with various psychosocial theories that link the beliefs to the behavior such as the Reasoned Action Approach [19]. This approach hypothesizes that if dentist evaluates target behavior, believes other dental colleagues support/practice the behavior, and believes in his/her ability to practice, the dentist’s motivation will be greater and dentist will be more likely to execute the target behavior.

Social desirability or the tendency to portray a culturally accepted self-image was found to be a moderator in many studies [17, 29]. However, our data showed that social desirability only impacted the reported changes in DIs’ descriptive norms. A possible explanation for the observed finding could be that a ceiling effect occurred on the descriptive norms of individuals who scored high on social desirability, which may prevent showing further effect of the intervention [30]. In contrast, only for individuals who had a low tendency to give socially desirable answers (score four or lower on the 10-item SDS), did the ISAC intervention have a significant effect on participants’ descriptive norms. The ceiling effect is a popular concept in health interventions and longitudinal studies [31, 32]. It would suggest that participants who had a high score on the descriptive norms pretest measurement would show relatively small improvements in post-test measurements [30]. Our current data did not show a considerable change in terms of the participants’ perceptions of their colleagues’ practice of COCE, who scored six or more on the 10-item SDS.

Dentists believed that the gender of dentist has an influence on performing oral cancer examinations and patient education. In other words, participants believed that female dentists practice more COCE than male counterparts [9, 24]. The linear mixed effects regression model showed that female dental interns performed more COCE practice than males at baseline and post-intervention. At the six-month follow-up, males then scored higher than females on the same outcome. A possible explanation for the gender differences in performing COCE could be related to the gender distribution of the participating dental interns. Although the intervention included the full population of dental interns who practiced in Jazan Dental School, the number of females was slightly higher than males throughout the intervention period. The recent development in the medical education following the Saudi 2030 vision in health encourages the inclusion of women in various fields [33]. Aside from this, the nature of dental practice motivates many women to pursue dentistry as their major [34]. The increasing number of women joining medical schools is not limited to Saudi Arabia. Other countries, such as the United States and Jordan have also reported an increased number of women [34, 35]. Evidence suggests that there may be even greater proportions of women dentists practicing in the future [36]. An alternative explanation could be related to the fact that female medical students in general perform better than males academically and clinically [37, 38]. Several studies have shown that females outperformed males in academic scores as well as in clinical skills assessments [34, 39].

The present study demonstrates a first step to quantitatively proceed with the findings from previous qualitative study aimed at testing the ISAC intervention usability among dental interns [24]. The intervention tested in this study was tailored to the needs of the target population which is a strong point of this study. Furthermore, it was the first to estimate the effect of a multi-component intervention on dentists’ practices in a routine working setting. The engagement of people from the target group in the planning and implementation phases of the ISAC was vital to the success of the intervention. For instance, the focus group discussions revealed a lack of dentists’ self-efficacy in detecting OC and in providing tobacco cessation counseling; this information was insightful and was considered when designing the intervention. Although the effect of the ISAC intervention does not differ between male and female DIs, this finding is inconclusive and should therefore be interpreted carefully. Based on the nature of our study, the estimated effect might have been too small to be detected. However, the findings from the present study can assist in estimating the statistical parameters to inform power calculations, as well as improving the validity and efficiency of consequent trials that test novel interventions. As we made use of a convenience sample of dental interns in Saudi Arabia, the generalizability of our results may be limited to populations that have similar characteristics to our sample, e.g., dentists in high-risk areas for OC. To facilitate the generalizability/transferability of the study, we thoroughly evaluated the process of developing, designing, pretesting, implementing, and evaluating the intervention and published it [40].

### Implications for Research

The intervention study utilized a pretest posttest without a control group. Therefore, future pragmatic research with a robust design (e.g., a multicenter randomized trial) is needed to test the ISAC’s effectiveness on a wider scale, to enhance long-term adherence to the ISAC by dentists, and to maximize implementation of the ISAC in a broader variety of healthcare settings: for instance, assessing the feasibility of recruiting and training other healthcare providers (e.g., family medicine doctors) and dental organizations to deliver the ISAC intervention.

### Implications for Practice

The ISAC intervention empowered dentists by addressing their beliefs and improved necessary skills to provide OC screening and support tobacco users to quit and communicate better with patients. The ISAC intervention had a high level of acceptance among the dentists, who reported that it was consistent with social values and useful in everyday clinical practice. In addition, dentists believed it helps to understand their central role in preventing and discovering OC at an earlier stage and the consequences of not screening on patients’ quality of life. The two workshops in tobacco-cessation counselling and patient communication skills offered dentists real-world experiences of using scientific methods, guided by trainers specialized in tobacco cessation and patient communication. The intervention had a profound impact on dentists’ consciousness, confidence, and professional capability to fulfill their social responsibility toward controlling the burden of OC in Jazan region.

The significant insights obtained from the dentists who participated in the ISAC intervention will give other dentists practicing outside JDS the confidence to negotiate and to push their organization leaders’ focus from only patients’ dental complaints to include an evidence-based intervention that serves the local community needs. To illustrate, after the initial experiments, a few participants discussed with their clinical directors about the importance of performing complete OC examinations and patient education in the Jazan region, in order to contribute to improving public health, in alignment with Saudi Arabia’s 2030 Vision.

The ISAC intervention covers major issues in dentists’ behavior found from the needs assessment, as it was shown that dentists were not informing patients about OC examination, not performing complete OC examinations, and not educating patient on OC risk factors, there was a lack of patient communication skills, and they did not connect patients to specialized services when they required advanced help. The findings from the ISAC effect evaluation revealed that the intervention was associated with successful improvements in terms of dentists’ cognition and behavioral skills. Dentists, dental organizations, and policymakers in areas at high risk of OC could benefit from the current intervention for the early detection and prevention of the disease.

## Conclusion

The findings from this study, reinforced by the theoretical and empirical evidence, confirmed that the ISAC intervention positively affected dentists’ cognitions and behavior toward oral cancer examinations and patient education practice, which favors the alternative hypothesis. The results showed promising effects of the intervention regarding patient communication skills, tobacco-cessation counseling and oral cancer examination skills. For the future implementation of the ISAC intervention, pragmatic research with a robust design is needed to test the effectiveness of the intervention on a wider scale.

## Data Availability

The original contributions presented in the study are included in the article. Further inquiries can be directed to the corresponding author: imoafa@jazanu.edu.sa.

## Abbreviations

*OC*: Oral cancer
*ISAC*: Inform, screen, advise, and connect
*DIs*: Dental interns
*IM*: Intervention mapping
*JDS*: Jazan Dental School
*CDD*: Community Dentistry Division
*COCE*: Comprehensive oral cancer examination score
